# Differential Induction of Ly6G and Ly6C Positive Myeloid Derived Suppressor Cells in Chronic Kidney and Liver Inflammation and Fibrosis

**DOI:** 10.1371/journal.pone.0119662

**Published:** 2015-03-04

**Authors:** Bastian Höchst, Julita Mikulec, Tania Baccega, Christina Metzger, Meike Welz, Julia Peusquens, Frank Tacke, Percy Knolle, Christian Kurts, Linda Diehl, Isis Ludwig-Portugall

**Affiliations:** 1 Institute for Molecular Medicine, University of Bonn, Bonn, Germany; 2 Institute of Molecular Immunology, Technische Universität München, Munich, Germany; 3 Institute for Experimental Immunology, University of Bonn, Bonn, Germany; 4 Department of Medicine III, University Hospital Aachen, Aachen, Germany; 5 Institute of Experimental Immunology and Hepatology, University Medical Center Hamburg-Eppendorf, Hamburg, Germany; Purdue University, UNITED STATES

## Abstract

CD11b^+^Gr1^+^ myeloid derived suppressor cells (MDSC) are known to be very potent suppressors of T cell immunity and can be further stratified into granulocytic MDSC and monocytic MDSC in mice based on expression of Ly6G or Ly6C, respectively. Here, using these markers and functional assays, we aimed to identify whether MDSC are induced during chronic inflammation leading to fibrosis in both kidney and liver and whether additional markers could more specifically identify these MDSC subsets. In an adenine-induced model of kidney inflammation/fibrosis suppressive Ly6G^pos^ MDSC were induced. The suppressive function within the Ly6G^+^ MDSC population was exclusively present in IFNγRβ expressing cells. In contrast, in chronic inflammation in the liver induced by bile duct ligation, suppressive capacity was exclusively present in the Ly6C^pos^ MDSC subset. Gene expression analyses confirmed the differential origins and regulation of those MDSC subsets. Additionally, depletion of MDSC in either kidney or liver fibrosis enhanced fibrosis markers, indicating a protective role for MDSC in organ fibrosis. Thus, our data demonstrate that during liver inflammation and kidney fibrosis MDSC with similar function arise bearing a distinct marker profile and arising from different cell populations.

## Introduction

Myeloid-derived suppressor cells (MDSC) are a heterogeneous population of cells with a myeloid origin. Murine MDSC are generally CD11b and Gr-1 positive [1–3] and can mediate suppression via several mechanisms (Arginase-1, iNOS, ROS) [4] MDSC are described to exert immunosuppressive function in cancer [10, 11], acute and chronic infections [12, 13]), under chronic inflammatory conditions [2], but also in autoimmune diseases [1]. Multiple inflammatory mediators such as IFNγ, TLR ligands [2], TNF [3], PGE_2_ [4, 5], S100 [6, 7], IL-1β [8] and IL-6 [9] have been described to induce, accumulate or activate MDSC, which then suppress T cell responses [10], modulate the cytokine expression by macrophages [11] or impair DC development [6]. Especially the role of IFNγ on the function and development of MDSC is discussed controversially. Whereas some publications show that the development of MDSC is IFNγ-dependent and that IFNγ is needed for the ROS or NO production [12, 13], other studies, in which MDSC development still occurred in IFNγR-deficient mice, suggest that IFNγ is not essential [10].

Organ fibrosis is a result of chronic inflammation and is accompanied by the infiltration of pro-inflammatory monocytes, macrophages, neutrophils and T cells. These inflammatory conditions go hand in hand with wound healing processes, which lead to continued replacement of dying parenchymal cells with connective tissue or extracellular matrix [14]. Organ fibrosis leads to severe functional damage of the organ and is one of the leading reasons for morbidity and mortality with growing prevalence in end-stage liver or kidney disease. During chronic inflammation many factors (e.g. IL-1β, TNF, IFNγ, DAMPs) are released, which may promote accumulation, activation or induction of MDSC in the inflamed organ [15]. These MDSC may then prevent immune‐mediated damage and reduce the harmful effects of prolonged inflammation by switching off pro-inflammatory immune cells. However, specific identification of MDSC in chronically inflamed fibrotic organs is challenging, as pro-inflammatory monocytes, neutrophils and macrophages, expressing similar markers and effector molecules but lacking suppressive function, also infiltrate the inflamed tissue. In addition to their suppressive function, MDSC can be subdivided into two major populations that either express Ly6C or Ly6G [1, 16] More specifically, monocytic Ly6C^pos^ MDSC express CD11b, Gr-1, Ly6C but no Ly6G and the granulocytic/neutrophilic Ly6G^pos^ MDSC express CD11b, Gr-1, Ly6G, but are low in Ly6C[15]. Many additional markers, such as B7H1, IL4Rα or IFNγRβ, are suggested to more specifically identify MDSC [17]. Myeloid cells stratified according to these markers can fulfil distinct suppressive functions in different diseases such as cancer, infection or autoimmunity [1, 18, 19]. However, it is not clear if these MDSC subpopulations play a suppressive role in organ fibrosis due to chronic inflammation.

In the kidney a suppressive role for MDSC has been described in renal cell carcinoma and renal transplantation ([20, 21]), but their role in kidney fibrosis has not been addressed so far. In the liver MDSC are known to accumulate in mouse models of acute immune-mediated liver injury ([22]) as well as in patients with chronic inflammatory liver disease, like hepatitis C ([23]), or hepatocellular carcinoma ([24]), which is thought to arise in part as a result of chronic liver inflammation and fibrosis ([25]). A well-established experimental animal model for liver fibrosis is bile duct ligation (BDL) in rodents, in which hydrophobic bile acid mediated liver injury leads to chronic inflammation, fibrosis and ultimately hepatic cirrhosis. In adenine-induced tubulointerstitial nephritis, excessive insoluble adenine causes tubular cell damage also leading to chronic inflammation and end-stage fibrosis. In this study, we aimed to identify the MDSC subsets that arise during chronic inflammation leading to fibrosis in both the kidney and the liver and further investigated whether additional markers could more specifically identify these MDSC subsets. Here, we describe that during adenine-induced tubulointerstitial nephritis suppressive capacity resides within the Ly6G^pos^ MDSC subset that also expresses IFNγRβ. In contrast, during chronic hepatic inflammation after bile duct ligation, suppressive capacity was exclusively present in the Ly6C^pos^ MDSC subset and did not correlate with IFNγRβ expression. These data indicate that during liver and kidney fibrosis suppressive MDSC with similar function, but different phenotype are induced.

## Material and Methods

### Mice, diet, treatments

C57BL/6J mice were bred in the central animal facility in Bonn according to the Federation of European Laboratory Animal Science Association guidelines and maintained under SPF conditions. Mice were fed an adenine-enriched diet (10g/5kg) (Sniff, Soest, Deutschland) to induce kidney fibrosis [26] Mice were injected with 10 μg LPS and 1 μg IFNγ every second day for 2–3 times to induce MDSC in the spleen [2]. Retinoic acid (Tretinoin, Roche, Grenzach-Wyhlen, Germany) was provided at 1g/liter in the drinking water of mice from day 7 onwards after bile-duct ligation or from day 7 on after the start of adenine feeding. This corresponds to a daily dose of approx. 0,5 mg/mouse. All efforts were taken to minimize suffering. Mice were sacrificed by cervical dislocation. All animal experiments were approved by the Animal Care Commission of Nordrhein-Westfalen (84–02.04.2013.A014 and 84–02.04.2013.A129).

### Bile duct ligation

Preoperatively, mice were injected with 5mg/kg carprofen. Mice were anesthetized by isoflurane inhalation during surgery. The abdomen was opened by a midline laparotomy and the bile duct was carefully mobilized and ligated with 5–0 silk. In sham-operated controls, the bile duct was mobilized but not ligated. The incision was closed with 5–0 silk. Mice were sacrificed at the indicated times after surgery and non-parenchymal liver cells were isolated and analyzed by flow cytometry.

### Isolation of non-parenchymal liver and kidney cells

Isolation of liver non-parenchymal cells was performed as described before [27]. Shortly, livers were perfused and mechanically dissociated. Non-parenchymal cells were collected from the interface after density centrifugation. Cells were analysed by flow cytometry or sorted using the gating strategy as indicated. Isolation from the kidney was performed as described earlier [28] Briefly, kidneys were perfused, mechanically dissociated and digested in RPMI 1640 (Gibco, Life Technologies, Darmstadt, Germany) containing collagenase and DNase. After letting tubular cells sediment for some minutes from homogenized cell suspensions the supernatant with non-parenchymal cells was taken to perform flow cytometry analysis or sort using the gating strategy as indicated.

### Generation of bone marrow-derived MDSC

Bone marrow was flushed from tibias of C57BL-6 mice and cultured in alpha MEM w/o L-Glutamine (Lonza, Belgium) supplemented with 10% FCS (PAA), 1mM sodium pyruvate (Biochrom AG), 2mM L-glutamine (Gibco, life technologies), 100U/ml Penicillin, 100μg/ml Streptomycin (Gibco, life technologies), 0,05 mM 2-mercaptoethanol (Gibco, life technologies) and 200 U/ml (= 40 ng/ml) CSF-2 (GM-CSF) (Peprotech, USA) in petri dishes. After 4 days of culture cells were analysed by flow cytometry or sorted for *in vitro* suppression assays.

### Real-time PCR

Liver and kidney tissue samples were homogenised and total RNA was isolated using the NucleoSpin RNA kit (Macherey-Nagel, Düren)). RNA was transcribed into cDNA using the High-Capacity cDNA Reverse Transcription kit (Gibco, life technologies) following the manufacturers instructions. Real-time PCR was performed using the SybrGreen PCR Master mix on a light cycler 480 instrument II (Roche, Switzerland) Primers used: α-SMA fw: 5′-TCCAGAGTCCAGCACAATACCAGT-3′, rv: 5′-TGACAGAGGCACCACTGAACC-3′, TGF-β fw: 5′-GCGGTCCACCATTAGCACG-3′, rv: 5′-GCTCGCTTTGTACAACAGCACC-3′. Collagen IV fw: 5′-TGGTGTGCACGAAGGA-3′, rv: 5′-GGCGGTACACAGTCAGACCAT-3′and vimentin fw: 5′-GCAAGGATTCCACTTTCCGTT-3′, rv: 5′-GCACCCTGCAGTCATTCAGA-3′.

### T cell suppression assay

MACS-purified CD8 T cells were labelled with 0.1μM carboxyfluorescein-succinimidyl-ester (CFSE) and stimulated with Dynabeads Mouse T-activator CD3/CD28 for T-Cell expansion and activation (life technologies, Darmstadt, Germany). Different subpopulations of sorted myeloid cells were cocultured with the T cells at the indicated ratios. Proliferation of T cells was analysed after 72h by flow cytometry and was based on the CFSE dilution.

### Flow cytometry and sorting

The phenotype of myeloid cells was determined by multi-colour flow cytometry using the following antibodies: anti-CD11b (M1/70), anti-Ly6C (4K1.4), anti-Ly6G (1A8), anti-CD11c (N418), anti-F4/80 (BM8), anti-I-A/I-E (M5/114.15.2), anti-CD34 (RAM34), anti-CD64 (X54–5/7.1), anti-CD86 (GL-1), anti-CD80 (16–10A1), anti- IFNγRβ (MOB-47), anti-IL-4Rα (I015F8), anti-CD62L (MEL-14), anti-B7–H1 (MIH-5) and CD45.2 (104). Antibodies were purchased from eBioscience or Biolegend. Dead cells were excluded with Hoechst-33258 (Sigma). All stainings were performed in the presence of 10 μg/ml Fc-Block (clone 2.4G2). Isotype matching fluorochrome-labelled antibodies were used as indicated. For the enumeration of total cell numbers per organ an equal amount of counting beads was added to each sample. Samples were acquired using an LSR Fortessa (BD Bioscience, Heidelberg, Germany), cell sorting was performed with Aria III (BD Bioscience, Heidelberg, Germany), and data was analysed using FlowJo software (TreeStar, Inc, Ashland, OR).

### Inflammatory gene expression analysis

Non-parenchymal cells were isolated from individual livers and kidneys 14 and 10 days after BDL and the start of adenine feeding, respectively. Cells were stained with antibodies against CD11b, Ly6G and Ly6C in the presence of Fc-block (clone 2.4G2). Cells were washed and subsequently sorted into CD11b+Ly6C+ and CD11b+Ly6G positive fractions from each organ. 20.000 cells were analysed for their inflammatory gene expression profile using the nCounter Mouse Inflammation Gene Expression CodeSet (Nanostring Technologies, Seattle, USA) according to the manufacturer’s instructions.

### Statistics

All experiments were performed at least three times with groups of 3 mice unless otherwise stated. Results are expressed as mean ± SEM. Statistical significance was calculated using ANOVA (*p≤0.05, **p≤0.01, ***p≤0.001).

## Results

### Ly6C and Ly6G positive myeloid cells accumulate during inflammation and fibrosis in liver and kidney

**Fig 1:**
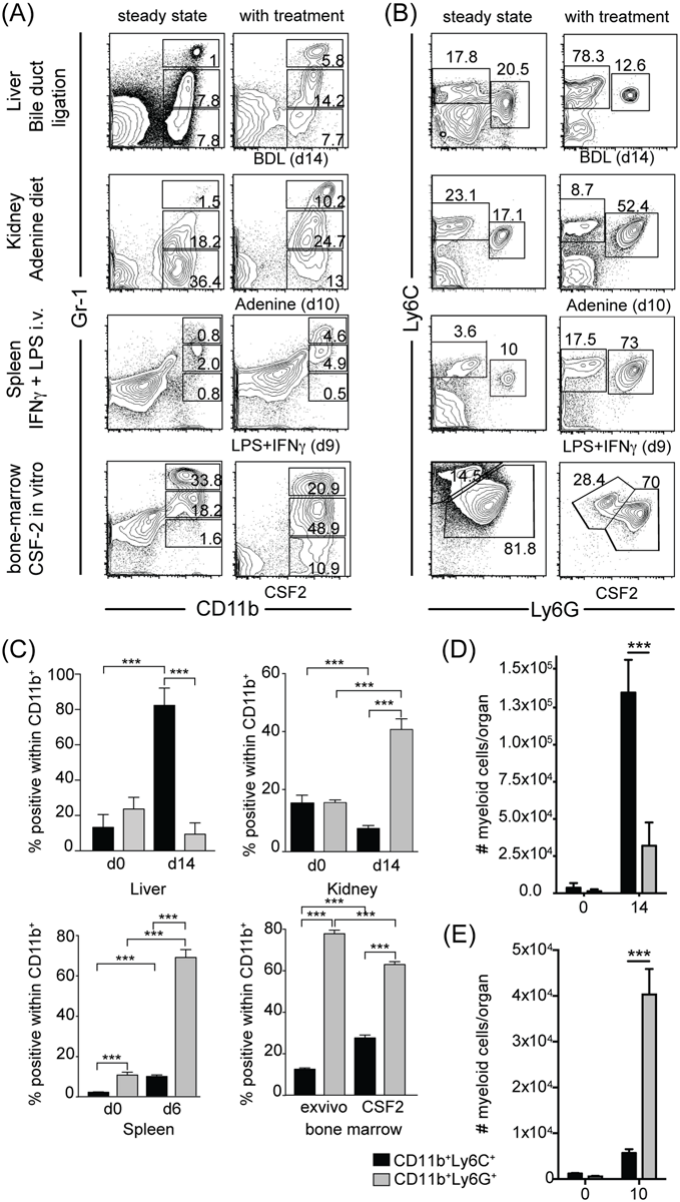
Differential distribution of monocytic and granulocytic myeloid derived suppressor cells within Gr-1 positive cells in liver and kidney inflammation and fibrosis. C57BL/6 mice underwent bile-duct ligation, were fed an adenine-enriched diet, or were injected i.v. with LPS/IFNγ. Furthermore, BM-MDSC were generated *in vitro* by culture of bone marrow cells with CSF2 (GM-CSF) (A, B). After the indicated times liver, kidney, spleen or bone marrow cells were isolated and analysed by flow cytometry. Histograms depict viable (Hoechst negative), non-parenchymal cells stained with CD11b and Gr-1 (A) or viable, CD11b^pos^ cells stained for Ly6G and Ly6C (B). Representative (A, B) and cumulative (C) data of 4 (liver, spleen) or 3 (kidney, bone marrow) independent experiments are shown (n>9). Absolute numbers of CD11b^+^Ly6C^+^ and CD11b^+^Ly6G^+^ meyloid cells in the liver (D) and kidney (E) at the indicated time-points after BDL or adenine-feeding, respectively. Data are depicted as mean +/- SEM. Significance was calculated by ANOVA. *p≤0.05, **p≤0.01, ***p≤0.001.

In order to characterise MDSC arising *in vivo* after chronic inflammation we induced liver fibrosis via bile-duct ligation (BDL) [29] and kidney fibrosis by feeding mice an adenine rich diet [26]. Furthermore, as a positive control, mice were either injected with LPS/IFNγ, as this has been shown to induce suppressive Gr-1 positive MDSC in the spleen, or *in vitro* generated BM-derived MDSC were used [2]. Although under steady state conditions, MDSC-like myeloid cells are present in the liver, kidney and spleen (Fig. 1A), upon BDL, adenine feeding or LPS/IFNγ treatment, the numbers of CD11b^+^Gr-1^int/high^ myeloid cells significantly increased in the respective organs (Fig. 1A). We then determined the relative contribution of monocytic myeloid cells (CD11b^+^Ly6C^high^Ly6G^neg^) and granulocytic myeloid cells (CD11b^+^Ly6C^int^Ly6G^high^) within the Gr-1 positive myeloid population (Fig. 1B, C). Both subsets were present in liver and kidney, even under steady-state conditions, but monocytic myeloid cells were significantly increased after BDL whereas granulocytic myeloid cells were enriched in fibrotic kidneys after adenine feeding (Fig. 1B, C). In the spleen of LPS/IFNγ-treated mice and in the bone-marrow cultures both monocytic and granulocytic myeloid cells were present, although the proportion of granulocytic myeloid cells was increased in both cases (Fig. 1B, C). Not only did the proportion of CD11b^+^Ly6C^+^ and CD11b^+^Ly6G^+^ cells increase in the liver and kidney, respectively, also absolute numbers of both cell types were increased substantially (Fig. 1D, E) Together, these data show that during liver and kidney fibrosis myeloid cells with a different MDSC phenotype accumulate. Furthermore, by separately analysing Ly6G and Ly6C expression, populations of MDSC-like myeloid cells reported to be Gr-1 positive, show differential accumulation depending on the anatomical location.

### Monocytic MDSC accumulate in the liver whereas granulocytic MDSC are induced in the kidney during organ inflammation and fibrosis

**Fig 2:**
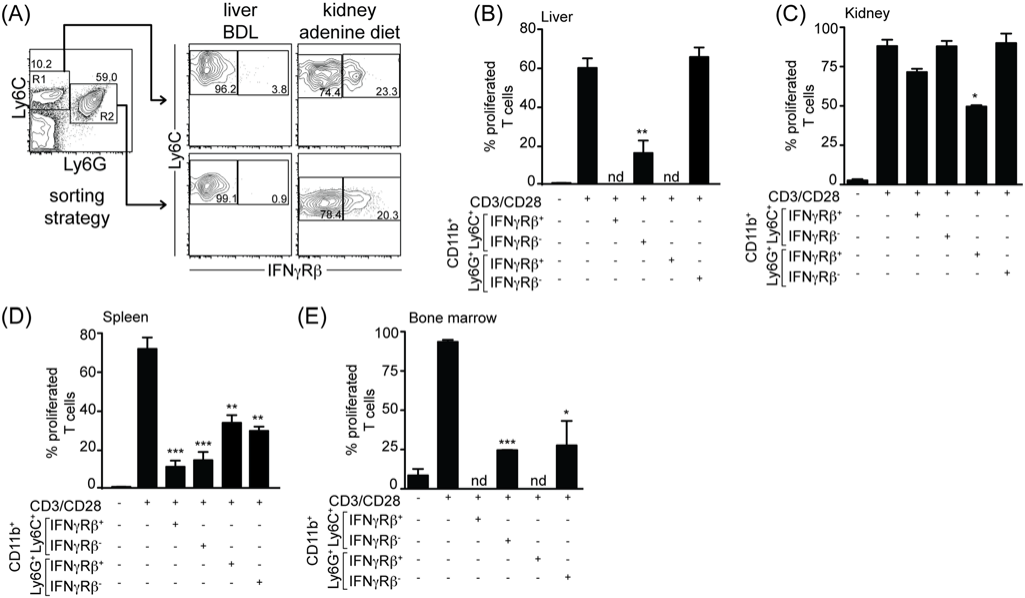
Suppressive capacity of monocytic and granulocytic MDSC subsets. C57BL/6 mice were treated as in Fig. 1. Gating strategy for sorting (A). At the indicated times myeloid subsets from liver (B), kidney (C), spleen (D) or in vitro bone marrow culture (E) were isolated and CD11b^+^ cells were sorted on the basis of their Ly6C or Ly6G expression and additional expression of IFNγRβ (A), yielding 4 separate subsets of CD11b^+^ myeloid cells. Naïve CFSE-labelled CD8 T cells were stimulated using αCD3/αCD28 coated beads and the different subsets of sorted myeloid cells were added at a 3:1 ratio (B-E). After 72h T cell proliferation was analysed by flow cytometry and the percentage of proliferated T cells is depicted (B-E). Cumulative data from 2 independent experiments are shown. Data are depicted as mean +/- SEM. Significance was calculated by ANOVA. *p≤0.05, **p≤0.01, ***p≤0.001. ND = not detectable.

Suppressive activity has been reported for both monocytic Ly6C^high^ expressing and Ly6G expressing myeloid cells [1, 16]. As we found that Ly6G and Ly6C expressing myeloid cells are induced with differential preference in organ fibrosis, cytokine injection *in vivo* and *in vitro* culture of bone marrow cells with CSF2 (Fig. 1), we analysed which of these subsets constituted MDSC and thus possessed suppressive capacity. As the development of MDSC is proposed to be IFNγ-dependent and that IFNγ is needed MDSC effector function [12, 13], we sorted CD11b^+^ myeloid cells according to their expression of Ly6C, Ly6G and IFNγRβ (Fig. 2A) and cocultured them with CFSE-labelled T cells in the presence of anti-CD3ε/CD28 coated beads. Interestingly, IFNγRβ positive Ly6C/G expressing myeloid cells were readily detected in the inflamed spleen and kidney, but not in the chronically inflamed liver after bile duct ligation or the bone marrow cultures. In bile duct ligated mice, Ly6C^pos^ myeloid cells possessed suppressive function (Fig. 2B), whereas in adenine-fed mice suppressive capacity was present within the renal Ly6G^pos^ myeloid subset (Fig. 2C), In both the *in vivo* LPS/IFNγ-induced and bone marrow-derived myeloid cells, suppressive activity was present in both Ly6G^pos^ and Ly6G^pos^ subsets (Fig. 2D, E). Moreover, in the kidney suppressive capacity was restricted to IFNγRβ expressing myeloid cells (Fig. 2C). In contrast, in the liver IFNγRβ was not expressed on suppressive myeloid cells (Fig. 2B), nor did the presence or absence of IFNγRβ on splenic myeloid cells influence their suppressive capacity (Fig. 2D). Thus, IFNγRβ expression does not seem to constitute a reliable or unique marker for the identification of functionally active MDSC. Gene expression analysis of a large panel of inflammatory genes revealed that the Ly6G^pos^ and Ly6C^pos^ MDSC from liver and kidney are not similarly regulated. Most genes that were differentially expressed by these two subsets, were either part of a Ly6C-signature (also present in Ly6C^pos^ cells from an adenine-fed kidney), or of a Ly6G-signature in both liver and kidney (S1 Fig.). A subset of genes was specifically regulated in kidney Ly6G^pos^ MDSC, of which several seem to be involved in the continuous attraction and activation of neutrophillic granulocytes/myeloid cells. Interestingly, neither liver nor kidney MDSC expressed arginase 1 (arg1) or iNOS (nos2) mRNA, indicating that suppressive function in both liver and kidney is not achieved via depletion of arginine or production of ROS. Together, these data indicate that MDSC accumulating during chronic inflammation and fibrosis in liver and kidney can originate from the monocytic or granulocytic myeloid subset, reflected by their gene-expression profile, depending on the location of induction. Additionally, IFNγRβ expression can be used to identify suppressive MDSC within the Ly6G^pos^ myeloid compartment in kidney, but not liver, inflammation.

### MDSC influence fibrosis progression in both liver and kidney inflammation

**Fig 3:**
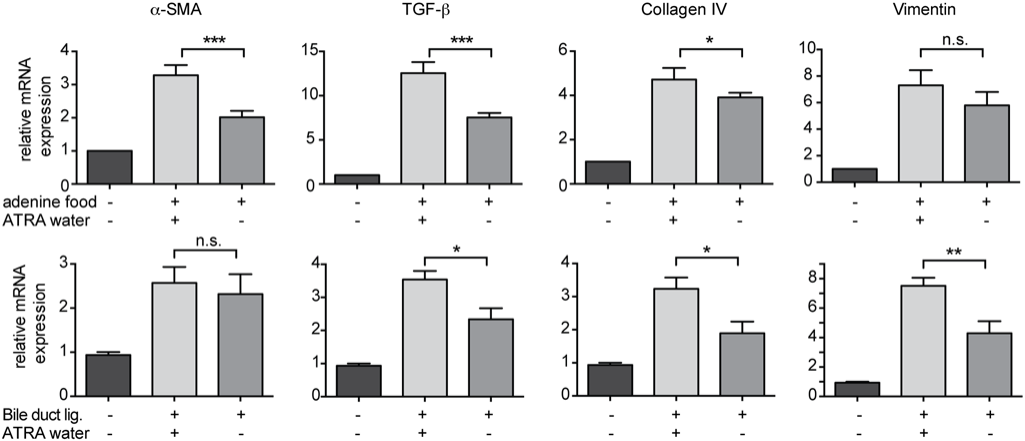
Fibrosis markers in the liver and kidney after all-trans-retinoic acid treatment. C57BL/6 mice underwent bile-duct ligation, were fed an adenine-enriched diet or as a control were left untreated. After 7 days mice were treated with 1g/L all-trans-retinoic acid (ATRA) in their drinking water (BDL: n = 7, Adenine: n = 4) or not (BDL: n = 8, adenine: n = 4)) for the remaining time until analysis. At day 14 (BDL and adenine feeding), total liver and kidney RNA was isolated for real-time PCR of fibrosis markers. Shown are mRNA expression levels for α-SMA, collagen IV, TGF-β and vimentin relative to the levels in non-treated mice (n = 3), which was set to 1. Data are depicted as mean +/- SEM. Significance was calculated by ANOVA. *p≤0.05, **p≤0.01, ***p≤0.001.

Although we find MDSC with potent *ex vivo* inhibitory function to accumulate during both liver and kidney fibrosis (Fig. 2), it was unclear whether the presence of MDSC would influence fibrosis progression. To test this, we treated bile duct ligated and adenine-fed mice with all-trans-retinoic acid (ATRA) in their drinking water or not, which is known to change MDSC functionality leading to their inability to exert suppressive function ([30]). As a control we sorted CD11b^+^Gr-1^pos^ cells from the liver of ATRA-treated bile-duct ligated mice and found that these did not posses suppressive capacity towards T cells in a proliferation assay (data not shown). In both bile-duct ligated and adenine-fed ATRA-treated mice, fibrosis markers like α-SMA, TGF-β, Collagen IV and vimentin were significantly increased (Fig. 3), indicating that the induction of MDSC during chronic inflammation dampens ensuing fibrosis in both the kidney and the liver.

### General MDSC specific surface markers not found

**Fig 4:**
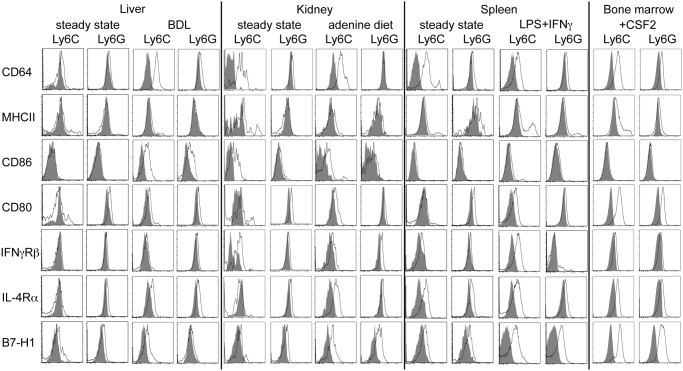
Surface marker expression on suppressive and non-suppressive myeloid subsets. C57BL/6 mice were treated and cells were isolated as in Fig. 1. Myeloid cells from non-treated mice served as controls (steady state). Flow cytometric analysis of surface markers associated with MDSC induction/function on CD11b^+^Ly6C^+^ and CD11b^+^Ly6G^+^ myeloid cells are depicted in the histograms. Specific staining: black lines. Isotype controls: filled grey. Representative data of 3 independent experiments is shown.

So far, one of the major difficulties in the study of MDSC function in inflammation-associated diseases is the absence of a reliable surface marker that is specifically expressed on myeloid cells with suppressive function. Although IFNγRβ expression defined Ly6G^pos^ MDSC in the setting of kidney fibrosis, we excluded the IFNγRβ as a general marker, since it was not universally expressed on monocytic or granulocytic MDSC (Fig. 4), as it was not expressed on liver-derived Ly6C^pos^ MDSC. Several other markers have been proposed as markers for MDSC in recent years. Thus, we performed a comprehensive phenotypic analysis to possibly identify a common additional marker for MDSC besides CD11b and Ly6G or Ly6C (Fig. 4 and S2 Fig.). Many of these already proposed markers are indeed expressed on several MDSC populations. For instance, IL-4Rα expression is induced during liver and kidney fibrosis and in the spleen after LPS/IFNγ treatment (Fig. 4). However, in liver and kidney the IL-4Rα is not only expressed on the suppressive subpopulation of myeloid cells, but also on the non-suppressive myeloid subpopulation, indicating that IL-4Rα expression cannot serve as a common marker for suppressive MDSC [33]. Furthermore, B7H1 expressed by MDSC can have a functional role in mediating suppression [17, 31]. In the liver, B7H1 expression was apparently restricted to the Ly6C^pos^ MDSC subset (Fig. 4). However, in the kidney Ly6C^pos^ and Ly6G^pos^ myeloid cells expressed B7H1, excluding B7H1 as a general MDSC specific marker. In order to clarify whether in principle liver and kidney MDSC are functionally similar or not we analysed mRNA expression levels of a panel of inflammation-associated genes (S1 Fig.). These analyses showed that these expression patterns were organ-independently either Ly6C- or Ly6G-associated but did not associate with suppressive function. Only a small panel of inflammatory genes were exclusively expressed in kidney Ly6G^pos^ MDSC, known to be involved in the chemotactic attraction of neutrophillic granulocytes (S1 Fig.). However, taken together these data do not identify a universal MDSC marker and supports the notion that such a marker may not exist, although within particular disease entities such additional markers can be identified.

## Discussion

Currently, the identification of suppressive MDSC in for instance murine tumour models is based on the co-expression of CD11b and Gr-1 on myeloid cells. Such CD11b^+^Gr-1^+^ myeloid cells are then tested for their suppressive capacity towards innate or adaptive immune cells to formally prove their MDSC status. Pro-inflammatory molecules such as IL-1β [32], IL-6 [9] and PGE_2_ [33] are thought to promote the induction of MDSC in tumours, whereas pro-inflammatory TNF signalling can drive the accumulation of MDSC in tumours [34]. Using CD11b and Gr1 surface molecules to identify suppressive myeloid cells, these cells are found in infection [35, 36] and autoimmunity [37]. In this study, we investigated whether in local chronic inflammation MDSC are induced to suppress ongoing inflammatory immune reactions and their resulting organ damage. We now demonstrate, using well-established models of kidney or liver inflammation and fibrosis, that during chronic inflammation, CD11b^+^Gr1^+^ myeloid cells expand. Further staining for Ly6C and Ly6G, both of which are recognised by anti-Gr1 antibodies, showed differential expansion of Ly6G^pos^ and Ly6C^pos^ myeloid cells. The first preferentially accumulate in the inflamed kidney, whereas the second accumulated significantly in the livers of bile duct-ligated mice, not only as percentage within CD11b^+^ myeloid cells but very prominently also in absolute numbers. In the inflamed kidney, suppressive MDSC expressed the IFNγRβ in addition to Ly6G. In contrast, in livers of bile duct ligated mice, suppressive MDSC expressed Ly6C but did not express IFNγRβ. In contrast, BM-derived MDSC did not express IFNγRβ at all, although these cells possessed suppressive capacity. Moreover, although IFNγ was necessary to induce MDSC in the spleen, expression of the IFNγRβ receptor was not required for suppressive activity of these splenic MDSC. The IFNγRα chain binds to IFNγ and the IFNγRβ chain transduces signals to the nucleus [38]. Although the IFNγRα chain is expressed highly on the membranes of B-, T- and myeloid cells, the IFNγRβ is only highly expressed only on the membrane of myeloid cells [39]. We now show that the IFNγRβ is not generally expressed on MDSC, which may explain the contrasting findings reported in literature on the importance of IFNγ for the induction and function of MDSC [10, 12, 13].

In order to classify MDSC induced in a specific organ, we decided to compare them directly to a defined MDSC “standard”, i.e. bone marrow derived and LPS/IFNγ induced splenic MDSC. Surprisingly, compared to BM and splenic MDSC, in the kidney and liver suppressive activity was only present in either Ly6G^pos^ or Ly6C^pos^ myeloid cells, respectively. We have recently reported that MDSC can be generated from monocytic cells by activated hepatic stellate cells (HSC) [36], indicating that monocytes arriving in the inflamed liver can locally be differentiated into suppressive MDSC during fibrosis when HSC become activated and differentiate into myofibroblasts. In the kidney such myofibroblasts, important for fibrosis progression, can arise from various subsets of renal cell populations, including fibroblasts, epithelial and endothelial cells [37], indicating that in different organs the cell population and their mechanisms of induction of MDSC may differ considerably.

Extensive phenotypic analysis of all myeloid cell subsets did not reveal a potential candidate for a liver MDSC specific marker. In tumours, IL4Rα expression not only correlates with suppressive function [40], but can also be used to specifically target MDSC [41]. However, in all of the conditions we tested, IL4Rα is expressed on both Ly6G^pos^ and Ly6C^pos^ subsets and cannot be used to specifically distinguish suppressive from non-suppressive myeloid subpopulations in kidney or liver inflammation and fibrosis. In livers of bile duct-ligated mice, B7H1 is expressed on the suppressive Ly6C^pos^ subset. Although in the kidney, spleen and bone marrow such a correlation cannot be detected, we and others have reported that hepatic stellate cells can induce MDSC [42, 43], which repress immune responses via B7H1 [31].

Gene expression analysis of a large panel of inflammatory genes revealed that both Ly6C^pos^ and Ly6G^pos^ MDSC were similarly regulated as their non-suppressive counterparts. For instance, Ly6C^pos^ MDSC in the liver express high levels of CCR2 and also produced the ligand CCL2, similar to for instance Ly6C^pos^ inflammatory monocytes ([44]). Ly6G^pos^ MDSC from the kidney did specifically induce the expression of several chemokines and chemokine receptors, involved in the attraction and functional modulation of granulocytes. This may reflect that MDSC in the kidney and liver utilize different signalling modes for further attraction of myeloid cells, which may then also be modulated into becoming MDSC.

Although we could not identify a general marker for MDSC, specific markers for MDSC, like the IFNγRβ in the kidney, may exist in specific disease entities. Thus, our study shows that during chronic inflammation leading to organ fibrosis in the liver and kidney myeloid-derived suppressor cells are efficiently induced capable of inhibiting potentially harmful immune responses.

## Supporting Information

S1 Fig Gene-expression analysis of Ly6C and Ly6G myeloid populations. Sorted cells from single livers and kidneys 14 and 10 days after bile-duct ligation or adenine-feeding. 20.000 cells were analysed. Average gene-expression of mean-centered data is shown for hepatic CD11b^+^Ly6C^+^ (n = 3), CD11b^+^Ly6G^+^ (n = 3) and renal CD11b^+^Ly6C^+^ (n = 1) and CD11b+Ly6G+ (n = 2) myeloid cells. High and low expression of individual genes is indicated by a colour code.
(TIF)

S2 Fig Additional phenotypical analysis of MDSC subtypes. Myeloid subsets isolated as in Fig. 1 were stained for various markers described to be associated with MDSC phenotype and/or function. Red squares indicate the suppressive populations.
(TIF)

S3 Fig Fluorescence minus one (FMO) control staining. FMO for IFNγRβ from a kidney after 10 days of adenine feeding before sorting. Gated on CD11b+Ly6G+ cells within a leukocyte gate without doublets.
(TIF)
