# Supplementary figures and images for: Differential Induction of Ly6G and Ly6C Positive Myeloid Derived Suppressor Cells in Chronic Kidney and Liver Inflammation and Fibrosis
Source: PLoS One. 2015 Mar 4;10(3):e0119662. doi: 10.1371/journal.pone.0119662 (PMC4349817; doi:10.1371/journal.pone.0119662)

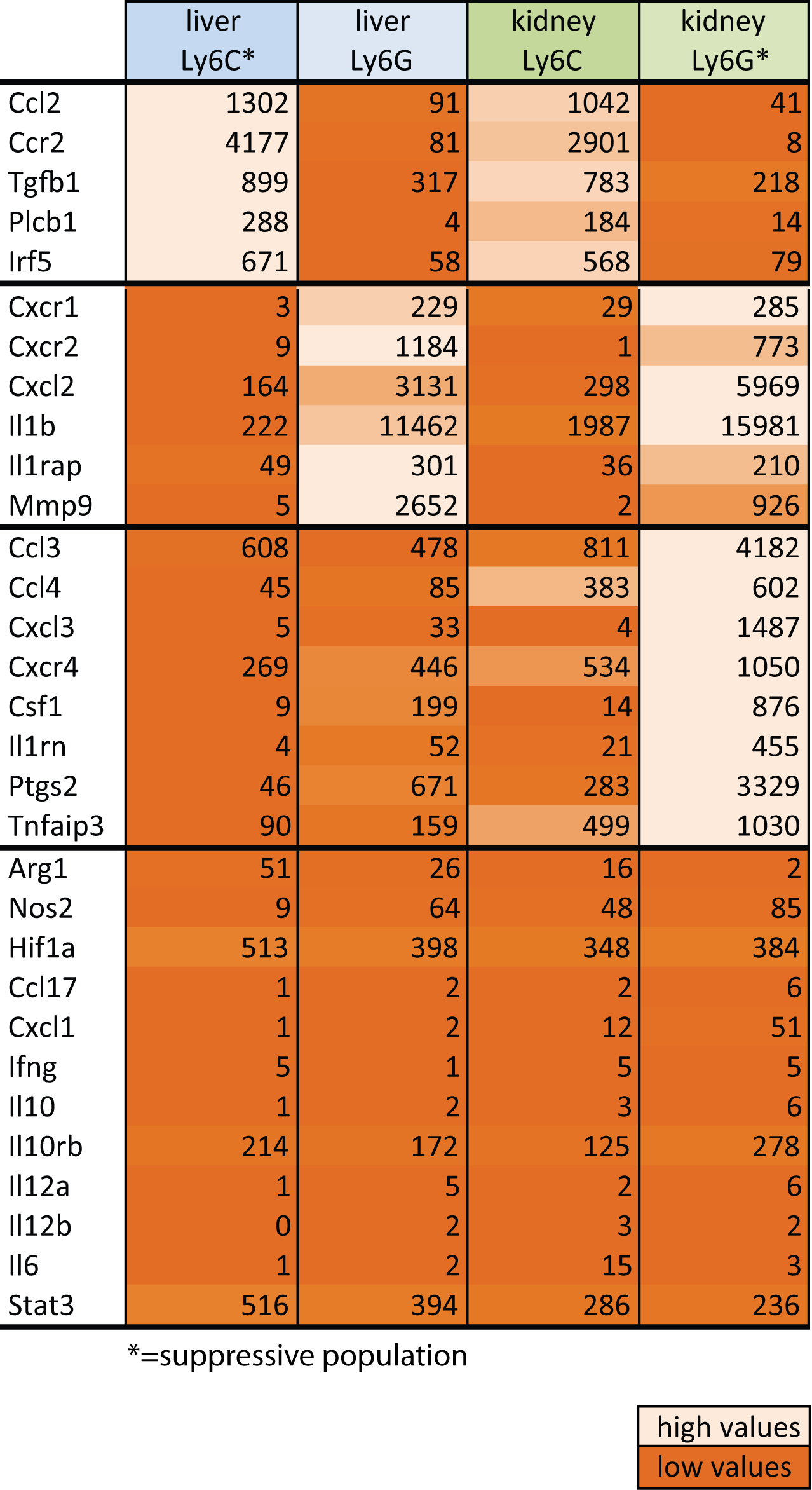

Supplement: S1 Fig — Sorted cells from single livers and kidneys 14 and 10 days after bile-duct ligation or adenine-feeding. 20.000 cells were analysed. Average gene-expression of mean-centered data is shown for hepatic CD11b+Ly6C+ (n = 3), CD11b+Ly6G+ (n = 3) and renal CD11b+Ly6C+ (n = 1) and CD11b+Ly6G+ (n = 2) myeloid cells. High and low expression of individual genes is indicated by a colour code. (TIF) [file pone.0119662.s001.tif]

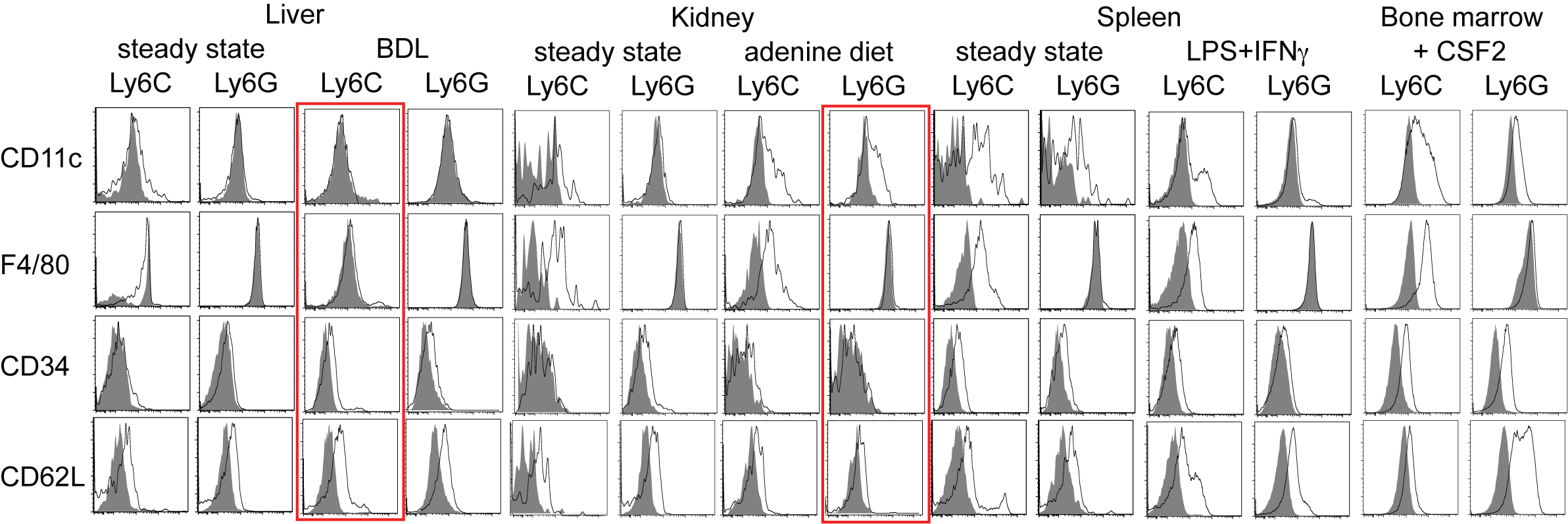

Supplement: S2 Fig — Myeloid subsets isolated as in Fig. 1 were stained for various markers described to be associated with MDSC phenotype and/or function. Red squares indicate the suppressive populations. (TIF) [file pone.0119662.s002.tif]

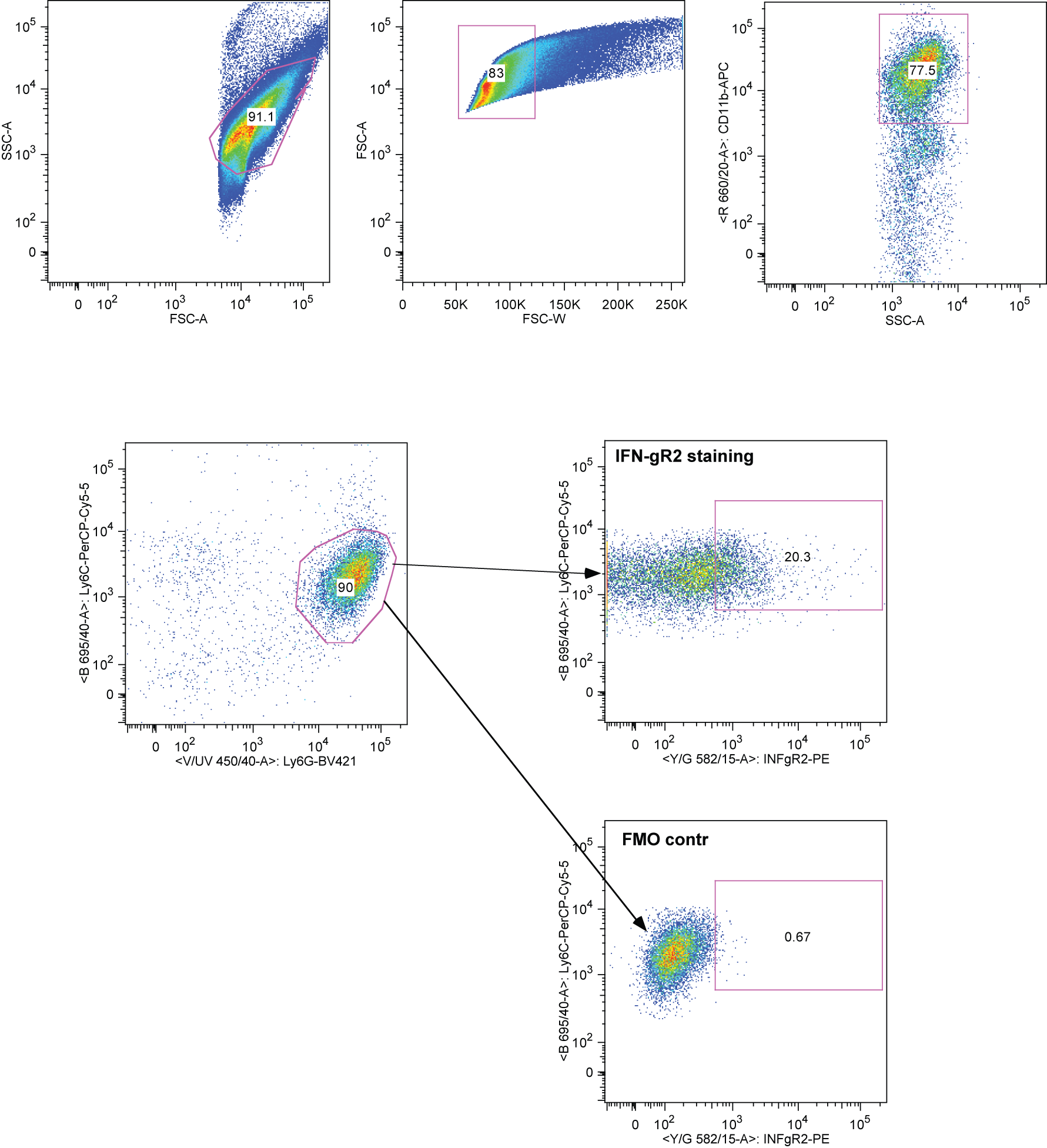

Supplement: S3 Fig — FMO for IFNγRβ from a kidney after 10 days of adenine feeding before sorting. Gated on CD11b+Ly6G+ cells within a leukocyte gate without doublets. (TIF) [file pone.0119662.s003.tif]
